# Running Promotes Transformation of Brain Astrocytes Into Neuroprotective Reactive Astrocytes and Synaptic Formation by Targeting Gpc6 Through the STAT3 Pathway

**DOI:** 10.3389/fphys.2021.633618

**Published:** 2021-05-21

**Authors:** Zhe Chen, Meng Gao, Yanlin Su, Pengran Liu, Binlei Sun

**Affiliations:** ^1^School of Physical Education & Sports Science, South China Normal University, Guangzhou, China; ^2^Department of Cardiothoracic Surgery, Xiangya Changde Hospital, Changde, China; ^3^Union Hospital, Tongji Medical College, Huazhong University of Science and Technology, Wuhan, China

**Keywords:** stroke, astrocytes, STAT3, Gpc6, A2 phenotype

## Abstract

Ischemic stroke is caused by cerebral ischemia upon the blockage of an artery, which results in a high disability rate. Little is known regarding the mechanism of astrocyte function in cerebral ischemia. We aimed to determine the effects of running on the transformation of astrocytes, and subsequent synapse formation. A study of middle cerebral artery occlusion (MCAO) after running *in vivo* showed that running can promote the transformation of astrocytes toward the neuroprotective phenotype. Our findings of oxygen-glucose deprived astrocytes *in vitro* after running revealed that these astrocytes transformed into the neuroprotective phenotype, and that the expression of STAT3 and Gpc6 was increased. We confirmed that mechanistically, running can target Gpc6 through the STAT3 pathway and then regulate the number of synapses. We concluded that running promotes synapse proliferation by polarizing astrocytes toward the neuroprotective phenotype and ultimately leads to nerve regeneration.

## Introduction

Apoplexy is brain tissue necrosis after ischemia, which affects related brain functions. It is now the second leading cause of death and the third leading cause of disability worldwide (Phipps and Cronin, 2020). Despite considerable constant effort, stroke treatment has not significantly progressed, and prevention remains the main strategy. In addition, the high cost and potentially serious complications of stroke treatment can cause patients to lose confidence in prevention.

Acute cerebral apoplexy is acute ischemic stroke caused by cerebral vascular occlusion. Early resumption of cerebral artery blood flow is important for the recovery of patients and one of the most important and effective treatment strategies that mainly comprise intravenous thrombolysis and other methods of vascular blood flow reconstruction (Phipps and Cronin, 2020). Early treatment, by means such as thrombolysis (Becattini and Agnelli, 2020), plays a central role in the functional recovery of patients. Although thrombolysis and other methods have significantly improved the treatment of and recovery from acute stroke, much room for further improvement remains, and new therapeutic models are needed. Remote ischemic adaptation is a systemic preventive treatment against ischemic stroke.

Astrocytes account for ∼30% of the total number of brain cells and are thus the most prevalent cell in the brain (Giovannoni and Quintana, 2020). They respond to acute cerebral ischemia by transforming their immature astrocyte cell populations into mature activated cells and driving them in various differentiation directions. The main identified types of transformed astrocytes are neuroprotective and neurotoxic (Zamanian et al., 2012). After brain injury, neurotoxic cells produce proinflammatory factors, such as IL-6, which induce inflammation that damages the structure and function of nerve cells (Liddelow et al., 2017). Astrocytes express high levels of calcium ions and mainly secrete neurotrophic factors, which play an important role in axonal growth and synaptic formation, including nerve axon growth, indicating that astrocytes affect the growth and development of nerve cells mainly through cell-to-cell interaction (Morizawa et al., 2017). After ischemia, astrocytes in brain tissues can improve the survival rates of nerve cells in various ways, by providing metabolic support, and secreting antioxidants and neuroprotective substances (Chen and Swanson, 2003).

The Janus kinase-signal transducers and activators of transcription (JAK-STAT) pathway is the main signaling mechanism for a wide range of cytokines and growth factors. Activated JAK phosphorylates STAT, which then forms a STAT dimer that enters the nucleus and binds to the promoter region, where it activates or represses gene expression. Evolutionarily conserved JAK- STAT signals allow phosphorylated STATs to cross-talk across many channels and interact with each other. This pathway is stimulated by many external environmental factors, and leads to increased cell plasticity (Aaronson and Horvath, 2002).

Proteoglycans comprise disaccharide complexes that form glycosaminoglycans (GAGs) that anchor to the cell membrane. The GAG chain is a key peptide that mediates interactions between proteoglycans and cell membranes. The proteoglycan glypican 6 (Gpc6), plays a key role in synaptic formation. Six mammalian glypican (GPC) genes are homologous to Dally and Dlp in fruit flies (Weksberg et al., 1996). The functional domains of glypicans do not significantly correlate. The three-dimensional structures of these proteins are similar and the localization of 14 cysteine residues is conserved. Additionally, insertion positions of the blocking chain are similar, but the numbers of insertion points in each glypican subtype differ. For example, Gpc3 and Gpc6 have two and three sites, respectively. Glypicans play key roles in axonal regeneration and synaptic formation, and can be regulated by Wnt/β-catenin, JAK/STAT3, and other signaling pathways (Bause et al., 2016). Among many roles, glycans interact with signal receptors and morphogenetic factors (Wnt, FGF, HH, and BMP) through their sugar chains.

Running benefits brain structure and function, elicits molecular and cellular effects, and alters mental states and consciousness. Marathon and regular running impacts glial and nerve cells in the brain. However, the effects of running on astrocytes in damaged brain tissues remain unclear. Therefore, we investigated the effects of regular running for a specific period on glial cells and neurons after cerebral ischemia in mice.

## Materials and Methods

### Ethics Statement

We obtained C57BL/6 mice from the Animal Experimental Center of Shandong First Medical University. All experimental procedures were conducted in accordance with the ethical requirements for animal experimentation at South China Normal University.

### Animals

Female mice were trained at 7.0 m/h for 6 days. Thereafter, the experimental group ran on a Model T408E electric horizontal treadmill (Diagnostic & Research Instruments Co., Taoyuan, Taiwan), at 10 m/min, 60 min/d, for 2 weeks. The control group did not run and were only placed on the running platform. All other conditions were the same.

We generated mouse models of middle cerebral artery occlusion (MCAO) using the Longa suture method. The MCA was occluded by inserting a thread into the carotid artery (CA) and extending it forward to the MCA bifurcation in 10 healthy female C57 mice (running and non-running groups, *n* = 5 each; weight, 25–30 g). We anesthetized the mice by administering an intraperitoneal injection of 10% chloral hydrate (Fisher Scientific Inc., Waltham, MA, United States). The necks of supine mice were disinfected, then the internal (ICA), external (ECA), and common (CCA) carotid arteries were separated on the right side via a median incision. The proximal and distal ICAs were clipped with sutures and vascular clamps. Sutures and bulldog forceps were placed at the proximal and distal ends of the incised ICAs, respectively. The CCA was incised (17–19 mm) again and sutured from the origin of the MCA to the proximal anterior cerebral artery (PACA). The sutures were fixed, then elapsed time was measured from the beginning of local cerebral ischemia. We disinfected and sutured incisions layer by layer. The mice were then returned to their cages.

Crawling mice turned right (tail-chasing phenomenon), and even fell rightwards. The right forelimb showed adduction and flexion, and the tail was raised, thus confirming MCA occlusion in the model. Control mice underwent skin incisions and vascular dissection without any suturing. The temperature of the mice was maintained at 37°C.

The MCAO was not permanent, and the ligation was released after 90 min of ischemia. The mice were euthanized by cervical dislocation at 24 h after MCAO.

Mice were processed for immunohistochemical assessment as follows. The mice were perfused via the ascending aorta with normal saline for 5 min to remove blood, and then perfused and fixed in 4% paraformaldehyde until the livers and tails became stiff. The animals were stored at 4°C for 2 h; then, the brains were excised and placed overnight in 4% paraformaldehyde at 4°C. Thereafter, the animals were dehydrated in 30% sucrose and fixed with neutral formalin for 24 h. After dehydration, brain tissues were paraffin embedded, sectioned, and sealed.

### Cells

Macerated brain tissues from 6 to 8-week-old, euthanized C57BL/6 mice were digested with 0.25% trypsin (Life Technologies Co., Grand Island, NY, United States) at 37°C for 30 min. The digest was filtered through 200-μm mesh, then the cells were centrifuged at 200 × g. Astrocytes purified by differential adhesion were cultured in Dulbecco’s Modified Eagle’s Medium (DMEM) (Gibco; Invitrogen, Grand Island, NY, United States) containing 8% fetal bovine serum (HyClone; GE, South Logan, UT, United States), which was changed every 3 days. We sub-cultured 85% of the cells as the third generation.

We extracted and cultured cortical neurons from the brains of 6–8-week-old C57BL/6 mice. Brain tissues were digested in 0.25% trypsin, then centrifuged. The cells were resuspended and seeded in culture dishes for 24 h. Neurobasal culture medium (Life Technologies Co.) was supplemented with 2% B27 basal culture medium (Life Technologies Co.) for 48 h. Thereafter, neurons were purified by adding Ara-C (Solarbio, Beijing, China).

Astrocytes were cultured with DMEM and 10% FBS. Neurons were cultured in neurobasal medium containing B27, L-Glu and NGF at 37°C under a 5% CO2 atmosphere. These were obtained from C57 mice after running or not for 6–8 weeks. The cells were extracted after MCAO and then exposed to oxygen-glucose deprivation (OGD) for 4 h to simulate ischemia *in vitro*. Briefly, the cells were cultured in DMEM without glucose at 37°C, under a 95% nitrogen/5% CO2 atmosphere and then cultured under normal conditions for 24 h.

### Immunohistochemistry

In brief, paraffin embedded tissue sections were dewaxed using a microwave, immersed in water, washed with phosphate-buffered saline (PBS pH 7.4; Gibco; Invitrogen), and incubated with 3% H_2_O_2_ at 27–32°C followed by primary antibody at 27–32°C, then coated with diaminobenzidine buffer and polymer. The sections were stained with hematoxylin, differentiated with hydrochloric acid, washed, sliced, dehydrated, sealed, then dried. Cells were visualized and photographed using an IX73 microscope (Olympus, Tokyo, Japan).

### Immunofluorescence

Immunofluorescence analysis showed that the area dominated by the cortical branch of the MCA was the lateral infarct area of the cerebral hemisphere. Briefly, sections with climbing cells were soaked in PBS, fixed, then incubated with 0.5% Triton X-100 (Beyotime, Shanghai, China) in PBS at 27–32°C. The sections were dried using absorbent paper, then normal goat serum (Solarbio) was applied to the sections, which were sealed at 27–32°C and placed at 4°C overnight. The sections were then immersed in PBS, dried using absorbent paper, incubated with diluted fluorescent secondary antibody at 27–32°C in a wet box, then washed with PBS. Nuclei were stained with DAPI (Solarbio)in the dark, then the sections were washed with PBST. Fluid on the climbing sheet was dried, sealed, and then sections were visualized using the IX73 microscope. Images were quantified using ImageJ software (National Institutes of Health, Bethesda, MD, United States).

### Western Blotting

The protein samples were centrifuged at 4°C, and tissues were homogenized in radioimmunoprecipitation assay buffer (Beyotime) containing phenylmethylsulfonyl fluoride and protease/phosphatase inhibitor (Beyotime). Thereafter, 5 × loading buffer was added to the protein supernatant, and total protein was quantified using BCA analysis kits (Beyotime). Non-specific protein binding on membranes was blocked with 5% skimmed milk for 2 h, then the membranes were incubated with primary antibody overnight at 4°C, followed by secondary antibody at 37°C for 60 min. Chemiluminescence was detected using Enhanced Chemiluminescence Kits and a gel imaging system (Invitrogen). The density of protein bands was determined using ImageJ software.

### Quantitative Real-Time Polymerase Chain Reaction (PCR)

Total mRNA was extracted from tissues with TRIzol reagent (Invitrogen) as described by the manufacturer, then RNA concentrations were measured. Complementary DNA (cDNA) was synthesized using reverse transcriptase and RT-qPCR Kits (Takara Bio Inc., Kusatsu, Japan). Quantitative real-time PCR (RT-qPCR) proceeded using a real-time PCR system (Bio-Rad, Hercules, CA, United States) with SYBR Green PCR premix (Takara Bio Inc.). Gpc6-specific primers were purchased from RiboBio Co., Ltd. (Guangzhou, China). Table 1 shows the primer sequences. Data were analyzed using the 2^–Δ^ ^Δ^ ^*Ct*^ method with U6/actin (RiboBio Co., Ltd.) for standardization.

**TABLE 1:** Primer sequences for PCR.

**Gene**	**Forward**	**Reverse**
U6	TGGAACGCTTCACGAATTTGCG	GGAACGATACAGAGAAGATTAGC
actin	CATCCGTAAAGACCTCTATGCCAAC	ATGGAGCCACCGATCCACA
Stat3	TGCACCTGATCACCTTCGAGAC	CCCAAGCATTTGGCATCTGAC
Gpc6	TCTGGTCAGCATTGCCCTACAC	TCAGCCCATCGTTCATGATCTC

### Chromatin Immunoprecipitation (ChIP)

We investigated STAT3 binding to target genes in astrocytes (4–5 × 10^7^/L) using ChIP kits (Cell Signaling Technology, Danvers, MA, United States) as described by the manufacturer. Cells were immobilized in 1% formaldehyde, cultured under OGD or normal (5% CO_2_, 37°C) conditions, then incubated with protease and phosphatase inhibitors. Separated chromatin was homogenized using a tissue grinder to extract DNA fragments of 200–1,000 base pairs. The DNA fragments were then incubated with an anti-STAT3 antibody (Cell Signaling Technology Inc.) that binds to magnetic beads. After separation and elution from the beads, reverse crosslinking proceeded as described by the manufacturer. The DNA was analyzed by quantitative PCR using primers.

### STAT3 siRNA

We purchased STAT3 siRNA from Changzhou Ruibo Biological Technology Co., Ltd. (Changzhou, China). Astrocytes (5 × 10^5^) were inoculated into 24 well plates to a cell density of 30–50%, then transfected as follows. Small interfering RNA (1.25 μL, 20 μM) was diluted in 30 μL of 1 x riboFECT^TM^ CP buffer. The riboFECT^TM^ CP reagent was gently mixed and incubated at room temperature for 0∼15 min to prepare transfection complexes that were gently mixed with an appropriate amount of complete culture medium without antibodies. The astrocytes were exposed to OGD, then incubated at 37°C for 24 h.

### Data Analysis

All results are expressed as means ± SD. Correlates were assessed using Pearson correlation coefficients. Unless otherwise indicated, data were analyzed using one-way analysis of variance, and Tukey and Dunnett tests were applied for multiple comparisons using Prism software (GraphPad Software Inc., San Diego, CA, United States). Values with *P* < 0.05 were considered statistically significant. Synapses in cultured nerve cells were counted using Image J software.

## Results

### Astrocytes Were Activated and Polarized to Neuroprotective Phenotypes After Running *in vivo*

**FIGURE 1:**
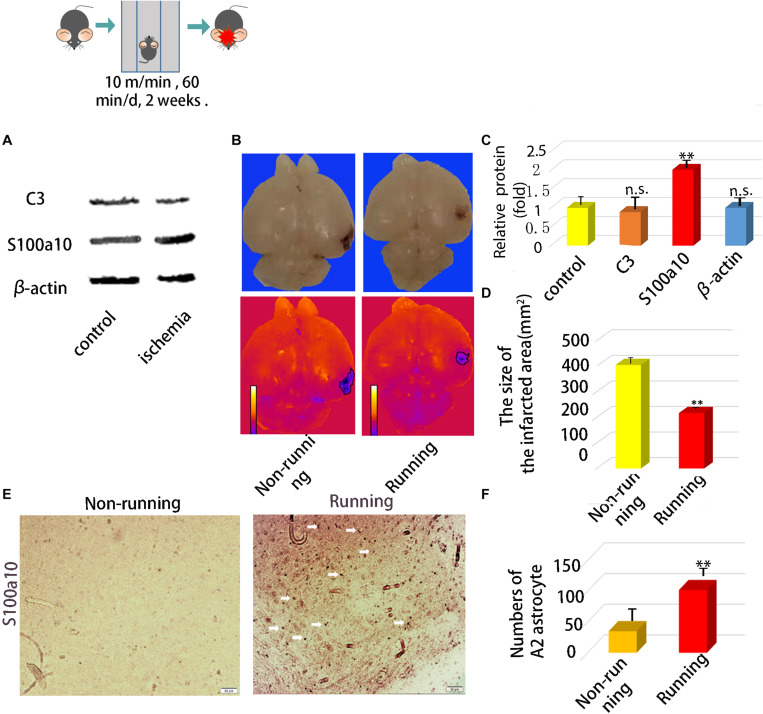
*In vivo* experiments confirmed that astrocytes were activated and polarized to neuroprotective type astrocytes after running. **(A)** The protein expression of A1 marker (C3) and A2 marker (S100A10) in ischemic area after ischemic injury. **(B)** Brain Anatomy of ischemic injury in mice. **(C)** Quantification of **(A)**. **(D)** The size of infarct area. **(E)** Immunohistochemical staining of brain tissue at the site of cerebral ischemia injury, S100a10 was staining with its antibody, Magnification × 200, scale bar 50 μm. **(F)** Numbers of A2 astrocyte after ischemia. Data were expressed as means ± SD, *n* = 5. ^∗∗^*P* ≤ 0.01.

After ischemia, the expression of C3 did not change significantly, but S100A10 increased significantly *p* < 0.05; Figures 1A,C). Infarct areas significantly decreased 0.52-fold in the running, compared with the non-running group (*p* < 0.05; Figures 1B,D). Immunohistochemical staining showed that neuroprotective astrocytes were significantly increased 2.91-fold in the brain tissues of the running, compared with the non-running group (*p* < 0.05; Figure 1E,F), and the cell volume was significantly increased.

### Astrocytes Induced by Running Were Polarized Toward Neuroprotective Phenotypes *in vitro*

**FIGURE 2:**
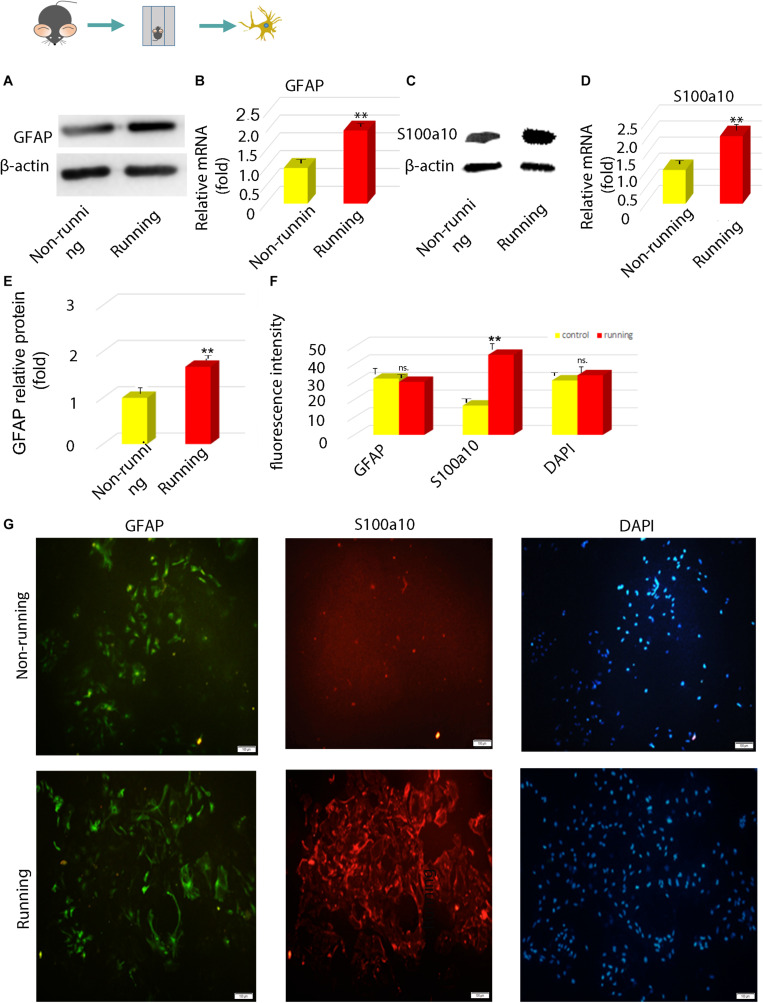
The polarization of astrocytes induced by running was confirmed to be toward neuroprotective astrocytes *in vitro*. **(A,C)** The expression of GFAP protein was detected by Western blot, β-actin is an internal reference protein. **(B,D)** The mRNA expressions of GFAP and S100A10 were detected by Q-RT-PCR. **(E)** Statistics of **(A)**. **(F)** Statistics of **(G)**. **(G)** The expression of GFAP and S100A10 in astrocytes was detected by immunofluorescence, Magnification, ×100. Scale bar, 100 mm. Data were expressed as means ± SD, *n* = 3. **P ≤ 0.01.

Immunofluorescence assays showed that cells were transformed into neuroprotective reactive astrocytes. Western blotting showed that GFAP and S100a10 protein levels were significantly increased 2.05-fold and 2.30-fold (*p* < 0.05; Figures 2A,C) in the transformed astrocytes compared with controls. Levels of GFAP and S100a10 mRNA expression determined by RT-PCR were increased 2.08-fold (*p* < 0.05; Figure 2B) and those of S100A10 were increased 2.11-fold (*p* < 0.05; Figure 2D) in the running, compared with the non-running group. Immunofluorescence intensity showed that S100A10 expression in astrocytes was 2.81-fold higher in the non-running, than the non-running group (*p* < 0.05; Figures 2E–G).

### STAT3 and Gpc6 Were Upregulated *in vitro*

**FIGURE 3:**
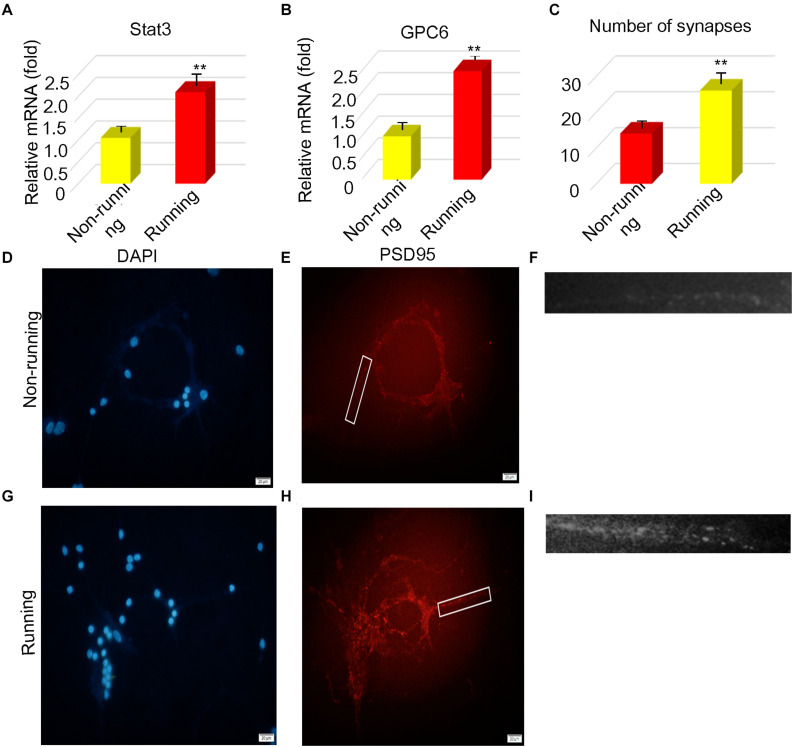
STAT3 and Gpc6 were upregulated *in vitro*. **(A,B)** The changes of STAT3 and gpc6 in astrocytes were detected by PCR. **(C)** The number of synapses was counted after astrocytes were cocultured with neurons. **(D–I)** The number of synapses was measured by immunofluorescence, The PSD95 of the postsynaptic membrane was stained and the nucleus was stained by DAPI, Magnification, ×400, scale bar 20 μm. Data were expressed as means ± SD, *n* = 3. **P ≤ 0.01.

We detected the STAT3 and Gpc6 genes that are associated with synapse formation in astrocytes after ischemia. The RT-qPCR findings showed that STAT3 and Gpc6 levels were 1.81- and 2.42-fold higher, respectively, in the running, than the non-running group (*p* < 0.05 for both; Figures 3A–C, respectively). We co-cultured astrocytes with neurons and found 1.86-fold more synapses in the running, than the non-running group (*p* < 0.05; Figures 3D–I).

### Expression of STAT3 Correlated With Numbers of Synapses

**FIGURE 4:**
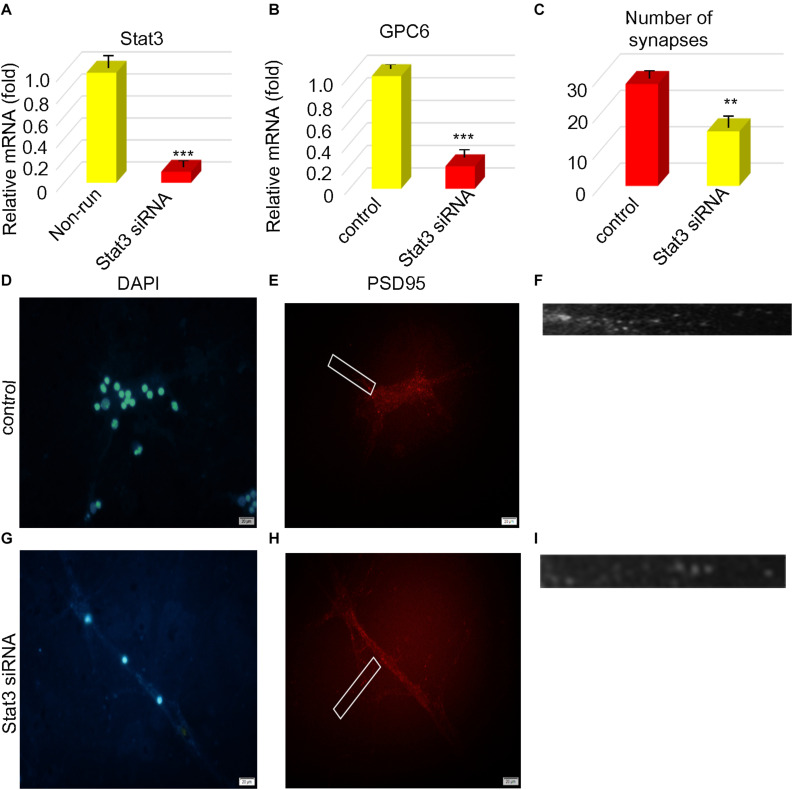
STAT3 correlated with the number of synapses. **(A,B)** The expression of STAT3 and gpc6 mRNA in astrocytes was detected by PCR after ischemia. **(C)** After STAT3 was interfered, the number of synapses was counted. **(D–I)** After STAT3 was disrupted, PSD95 was detected by immunofluorescence the nucleus was stained by DAPI, Magnification ×400, scale bar 20 μm, *n* = 3. Data were expressed as means ± SD, *n* = 3. **P ≤ 0.01, ***P ≤ 0.001.

**FIGURE 5:**
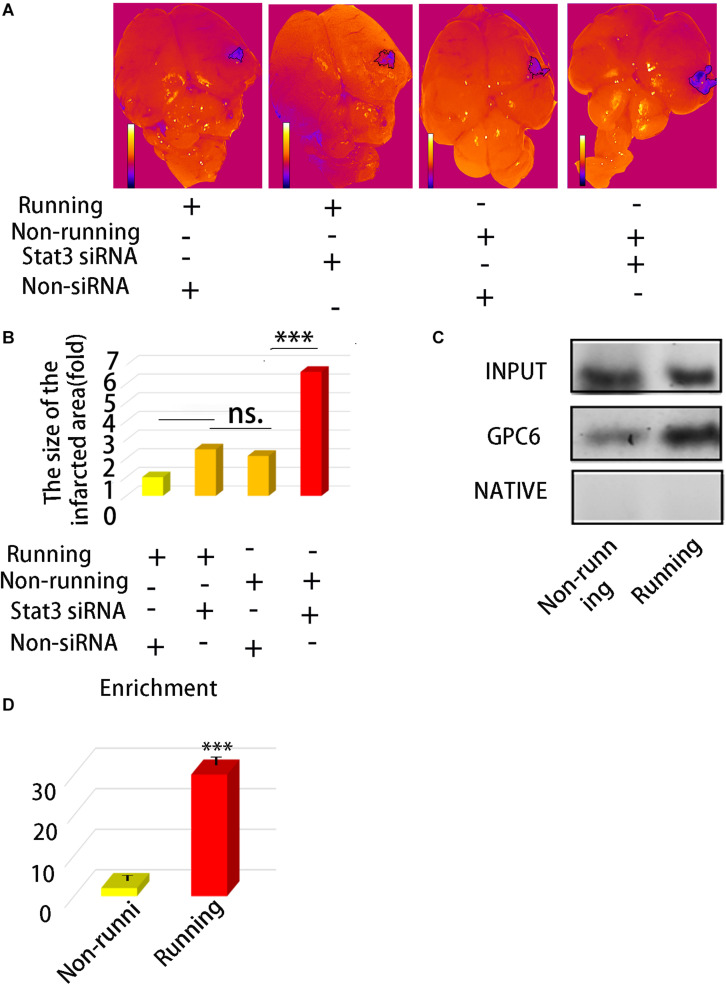
**(A)** Morphologic map of infarct area. **(B)** Statistics of **(A)**. **(C)** ChIP was used to detect the direct binding of STAXT3 with gpc6 promoter. **(D)** The enrichment efficiency of STAT3 combined with gpc6 promoter. Data were expressed as means ± SD, *n* = 3. ***P ≤ 0.001.

We explored the relationship between the number of protrusions and STAT3 expression. We found that STAT3 expression positively correlated with the number of synapsesin astrocytes stimulated with STAT3 siRNA compared with the unstimulated controls (Figure 4A). The expression of Gpc6 also decreased 0.20-fold (Figures 4B,D–I) compared with the control group (*p* < 0.05). We inhibited STAT3 expression in astrocytes using *STAT3* siRNA and found that STAT3 was decreased 0.12-fold and the number of synapses were decreased 0.54-fold compared with the control group (*p* < 0.05; Figure 4C). The infarct area was 2.49-fold higher in the running group with, than without siRNA (*p* < 0.05), but did not significantly differ between the running group with siRNA and the non-running group. The infarct area of the non-running group with siRNA was 6.64-fold larger (*p* < 0.05) that that of the running group without siRNA, and 3.11-fold larger than that of the non-running group without siRNA (*p* < 0.05; Figures 5A,B).

### STAT3 Directly Binds to Gpc6 to Promote the Expression of GPC

We found using ChIP assays that STAT3 directly bound to *Gpc6*. The enrichment efficiency of Gpc6 was enhanced 29.14-fold by STAT3 in the running group compared with the control (*p* < 0.05; Figures 5C,D) and the non-running group, and that this promoted *Gpc6* expression.

## Discussion

This study aimed to determine whether running can increase synaptic regeneration after cerebral ischemia. Running can significantly improve memory (Jacotte-Simancas et al., 2015; Duzel et al., 2016; Voss et al., 2019). Accordingly, we investigated the effects and potential benefits of running on brain injury caused by ischemic stroke. The stress- induced proliferation of astrocytes is accelerated during the acute phase after brain damage, due to ischemia and hypoxia, and astrocytes produce some cytokines that are beneficial to synaptic growth (Morizawa et al., 2017). However, astrocytes also produce cytotoxic factors such as chondroitin sulfate proteoglycans during the subsequent chronic phase, and form a glial scar that prevents nerve regeneration and the formation of synaptic connections (Sharma et al., 2012; Raposo and Schwartz, 2014). However, consensus has not been reached as to whether running preconditioning can reduce the secretion of harmful factors and increase the secretion of beneficial molecules during the chronic phase.

We initially conditioned the mice with running, then established models of cerebral ischemia and hypoxia. We found that the number of synapses significantly increased in the running, compared with the non-running group. Running can promote the formation of synapses. These results are consistent with those of Gyorkos et al. (2014), and support the notion that running can improve synapse repair after ischemia and hypoxia.

We also investigated how movement regulates synaptic growth. We analyzed extracted astrocytes and neurons and found a significant increase in astrocyte STAT3 levels after running. Peak protein levels were slightly greater during the acute, than the chronic phase of ischemia. However, the level was significantly greater in the running, than the non-running mice. These results suggested that STAT3 levels increase after running and upregulate STAT3 threshold levels in astrocytes in response to ischemia and hypoxia damage. Although some STAT3 is utilized in astrocytes after running, levels of pSTAT3 were still greater in the running, than the control mice. Studies have found that STAT3 promotes nerve and tissue regeneration, and plays important roles in the nervous system and other tissues. Our results confirmed that STAT3 promotes astrocyte regeneration after brain injury.

Since STAT3 expression was significantly increased in astrocytes, we screened genes that affect synapse formation. We found that Gpc6 is a direct downstream binding factor of Stat3. That is, phosphorylated STAT3 directly binds to the promoter region of Gpc6 to activate the transcription and expression of GPC6, which is an important protein associated with synapse formation.

Regular running can improve physique, reduce hypertension (Dimeo et al., 2012), improve diabetes (Riddell et al., 2017; Hamasaki, 2018), and alleviate other diseases. Running plays an important role in glucose and lipid metabolism. It can also regulate abnormal circulatory blood flow (Carter et al., 2018), improve vascular endothelial function (Guo et al., 2009), reduce blood viscosity (Kumar et al., 2009), improve cardiovascular diseases, and affect neurological diseases.

The effects of running are quite obvious in the nervous system, and in degenerative diseases of the nervous system such as Alzheimer disease (Valenzuela et al., 2020), Parkinson disease, multiple sclerosis, amyotrophic lateral sclerosis, and Huntington chorea (Rosenberg and Yang, 2007; Belviranlı and Okudan, 2018). Diseases caused by inflammation of the nervous system have obvious effects. Running can upregulate brain-derived neurotrophic factors to prevent the occurrence of neurodegenerative diseases (Lin et al., 2019). Studies have shown that consistent running for a specific period can reduce the severity of seizures (Rosenberg and Yang, 2007) in rats. Stroke protection also requires time. Running for 2 weeks can reduce brain damage after stroke, whereas running for 1 week or less will not have this preventive effect.

A series of pathological changes occur after a stroke. The first is ischemia, which leads to obstacles in the blood-brain barrier, the formation of vasogenic edema, bleeding, and finally changes in the brain microenvironment. Many factors can improve the brain microenvironment, for example, matrix metalloproteinases can reduce edema (Belviranlı and Okudan, 2018; Mamtilahun et al., 2019) and blood-brain barrier defects. Tumor necrosis factor can also increase ERK and other molecules to reduce edema, and aquaporin also plays an important role in the occurrence of edema (Mamtilahun et al., 2019). All these factors can be regulated by running. Our findings showed that running regulates the proliferation of synapses through STAT3, which in turn changes the microenvironment of the brain.

Central nervous regeneration is an important challenge for neurology. At present, a close relationship between running and nerve regeneration has been established. Running can increase the number of connections in the hippocampus of mice that result in improved short-term and spatial memory (Allen et al., 2012). Running can also increase insulin-like growth factor and promote the regeneration of hippocampal neurons in mice, reduce the occurrence of neural aging (Bouzid et al., 2015) and maintain neuronal activity (Lines et al., 2020), which might explain why it induces astrocyte transformation into the neuroprotective phenotype. However, this phenomenon needs further study.

Transient ischemia can promote metabolic changes in astrocytes mediated by calcium ions (Shiratori-Hayashi et al., 2020), as mitochondria are released to nerve cells to enhance their survival. It can also play a neuroprotective role through the regulation of glutamate (Mahmoud et al., 2019). High levels of ATP receptors (Gibbs et al., 2011) are also expressed in astrocytes. Antagonizing ATP receptors can prevent neuronal damage, suggesting that they can assume more than one state (Zamanian et al., 2012). That is, after activation, astrocytes can transform into many forms with various functions, such as A1 and A2 astrocytes.

Astrocytes can secrete many useful molecules such as fibroblast, glial derived nerve (Ohta et al., 2004), and vascular endothelial (Guo et al., 2009) growth factors, that can promote the growth and survival of neurons, and reduce apoptosis.

Reactive astrocytes play important roles in scar formation and immune regulation after injury. In addition to microglia (Liddelow et al., 2017), astrocytes play an important role in immune regulation of the nervous system, such as the phagocytosis of nerve fragments (Morizawa et al., 2017), which are conducive to the formation of synapses. They also regulate inflammation and upregulate TGF-β (Zhang et al., 2018), thus affecting scar formation.

The effects of running on the brain are mainly manifested at the molecular and cellular levels, and on brain structure and function, as well as mental state and consciousness. Here, we mainly analyzed the effects of running on the brain tissues at the molecular and cellular levels. That running confers positive effects on brain injury (Jacotte-Simancas et al., 2015) has been established, but few studies have explored the effects of running on synapses of neurons mediated via astrocytes. Our findings of the STAT3-GPC6 axis provide a new perspective for neurology and rehabilitation that should ultimately help to find a cure, or at least minimize risk of stroke. We intend to explore the relationship between brain morphology and molecular pathways, which is an important way to study consciousness, and the relationship between STAT3/Gpc6 and human memory.

## Data Availability Statement

The original contributions presented in the study are included in the article/supplementary material, further inquiries can be directed to the corresponding author/s.

## Ethics Statement

The animal study was reviewed and approved by the South China Normal University.

## Author Contributions

ZC conceptualized the study design and directed the project. ZC and MG performed all the experiments. YS reviewed the data. BS and PL analyzed, reviewed, and interpreted the data. All authors contributed to the manuscript revision, read, and approved the submitted version.

## Conflict of Interest

The authors declare that the research was conducted in the absence of any commercial or financial relationships that could be construed as a potential conflict of interest.
